# Telepharmacy during COVID-19: A Scoping Review

**DOI:** 10.3390/pharmacy9040183

**Published:** 2021-11-11

**Authors:** Elizabeth J. Unni, Kanchita Patel, Isaac Rex Beazer, Man Hung

**Affiliations:** 1 Department of Social, Behavioral, and Administrative Sciences, Touro College of Pharmacy, New York, NY 10027, USA; 2 Class of 2024, Touro College of Pharmacy, New York, NY 10027, USA; kpatel49@student.touro.edu; 3 Class of 2022, College of Pharmacy, Roseman University of Health Sciences, South Jordan, UT 84095, USA; ibeazer@student.roseman.edu; 4 College of Dental Medicine, Roseman University of Health Sciences, South Jordan, UT 84095, USA; mhung@roseman.edu

**Keywords:** telepharmacy, scoping review, COVID-19, implementation, challenges, strategies, innovative methods

## Abstract

The objective of this scoping review is to summarize the implementation of telepharmacy during the surge of COVID-19. This review will focus on answering four questions: During the COVID-19 pandemic, (1) what were the various telepharmacy initiatives implemented? (2) what were the challenges faced when implementing telehealth initiatives? (3) what were the strategies used by pharmacies to overcome the challenges, and (4) what were some of the innovative methods used by pharmacies to implement telepharmacy? A literature search was conducted to include publications post-March 2020 about telepharmacy implementation via PubMed Central database and Google searches. All articles were examined for inclusion or exclusion based on pre-determined criteria. A total of 33 articles were reviewed. The most commonly observed telepharmacy initiatives were virtual consultations, home delivery of medicines and patient education. Limited access to technology and lack of digital access and literacy were major barriers in the implementation of telepharmacy. New protocols were developed by healthcare systems and regulations were relaxed by countries to accommodate telepharmacy. Pharmacies that successfully implemented telepharmacy overcame these challenges through patient and pharmacist education. The review also revealed the steps that can be taken by pharmacy organizations, payers and entrepreneurs in leveraging the convenience of telepharmacy.

## 1. Introduction

A coronavirus identified as SARS-CoV-2, was discovered in December 2019 in Wuhan, China. Thereafter, the virus continued to spread and its infectious impact was seen across the world. To date, according to the World Health Organization (WHO), there have been over 203 million confirmed cases of Coronavirus Disease 2019 (COVID-19) and over 4 million deaths globally [1]. Initially, mitigation strategies such as social distancing and wearing a mask were implemented in efforts to reduce the spread. Shortly after, research and development began the process to authorize an emergency use of vaccines to help protect against the virus. However, those that were infected with COVID-19 most often experienced respiratory symptoms such as a cold, cough, and shortness of breath [2]. 

As the highly transmissible and virulent virus expanded in the United States before the release of vaccine in 2020, healthcare facilities began to experience a surge in the patient population who tested positive for COVID-19 and became burdened by increased rates of hospitalization [3]. Elective surgeries and noncritical medical services were postponed or limited [4]. Anxiety and fear were widespread, especially among those who needed to seek healthcare for non-COVID-19 emergencies [5,6,7]. Patients avoided seeking hospital care due to stay-at-home orders or fear of the rising contagion [8].

The use of telehealth was promoted during the COVID-19 pandemic to combat this situation. Telehealth is defined as the “use of electronic information and telecommunication technologies to support and promote long-distance clinical health care, patient and professional health-related education, public health and health administration” [9]. Telepharmacy is defined as a method used in pharmacy practice in which a pharmacist utilizes telecommunications technology to oversee aspects of pharmacy operations or provide patient-care services [10]. Telepharmacy operations and services include drug review and monitoring, dispensing, sterile and nonsterile compounding verification, medication therapy management, patient assessment, patient counseling, clinical consultation, outcomes assessment, decision support, and drug information [10]. Remote healthcare services during the pandemic have demonstrated benefits for the healthcare system and improving public health [11]. Access to telehealth services allowed for increased social distancing and reduced potential infectious exposures. In addition, the strain on healthcare facilities was reduced by minimizing patient demand to come into facilities [11].

Pharmacies were no exception to COVID-19′s impact. Upon declaration of the pandemic, they began to make swift changes in order to make safety for both patients and pharmacy employees a priority. Pharmacies were also not immune to the economic stresses imposed by government-mandated lockdowns. Many had to reduce staffing, take less compensation for the services provided, or close stores [12,13]. Furthermore, the pandemic created many drug shortages [12,14], making it difficult for some pharmacies to provide affordable options for their patients. Alternative options are the often used mechanism when there is a drug shortage, and the alternatives are not always the most cost effective, resulting in increased cost to the patient and healthcare system [15]. 

In addition to the economic challenges, there were also logistical complications regarding safety measures. Required social distancing limited the ability of patients to physically visit pharmacies; reduced staffing and transition of pharmacists to remote work further limited pharmacist–patient interactions [12]. Telepharmacy was soon recognized as a tool that could overcome many of the challenges presented by the pandemic while still providing quality patient care. However, telepharmacy has historically been difficult for many pharmacies to implement due to lack of appropriate legislation and reimbursement [16,17]. The advent of COVID-19 accelerated changes that would make telepharmacy a reasonable option. In the United States, relaxation of the Health Insurance Portability and Accountability Act (HIPAA) regulations [18] allowed pharmacies to utilize inexpensive teleconferencing platforms, such as Zoom or Skype, which would have otherwise been noncompliant with privacy standards. Additionally, emergency legislation allowed pharmacists to perform COVID-19 related activities such as COVID-19 testing, vaccinations and telepharmacy regardless of individual state laws [18,19]. Though these changes made telepharmacy more accessible from legal and monetary perspectives they are not permanent, nor did they provide guidance for implementation. 

As healthcare providers and patients are identifying the advantages of telepharmacy, even after the pandemic is under control, there is the potential for telepharmacy to continue. Though there are publications about the benefits of using telepharmacy and expert opinions and reviews before COVID-19, the pandemic accelerated the need for implementing telepharmacy due to social distancing and quarantine requirements. Few pharmacies that implemented it during the pandemic have reported it as case reports. Additionally, there were some opinion articles about telepharmacy that were published during the same time. Thus, the goal of this article is to review available literature during the surge of COVID-19 to summarize the implementation of telepharmacy to provide future guidance. The review examined all different types of pharmacies such as community, hospital, ambulatory pharmacy, etc. Specifically, this article will answer four questions: During the COVID-19 pandemic, (1) what were the various telepharmacy initiatives implemented? (2) what were the challenges faced when implementing telepharmacy initiatives? (3) what were the strategies used by pharmacies to overcome the challenges? and (4) what were some of the innovative methods used by pharmacies to implement telepharmacy?

## 2. Materials and Methods

A search of the literature was conducted in early Spring in 2021 and it was updated in Summer 2021 to include publications post March 2020. The key words used when searching included “telemedicine”, “telehealth”, “pharmaceutical care services”, “medication review”, “adherence counseling”, “community pharmacy”, “ambulatory setting”, “inpatient or hospital setting”, and “during COVID-19”, and a combination of these key words. The exclusion criteria were if the publication was prior to March 2020, if the studies were not published in English, did not have references to pharmaceutical care or did not have relevance to telepharmacy. The search was conducted via PubMed and Google search. All the articles identified were initially examined by authors K.P. and I.R.B. for inclusion. Once the articles were identified, they were then evaluated to answer the four questions stated in the objectives by two authors (K.P. and I.R.B.) individually. The findings were later discussed by the authors to reach a consensus. If no consensus was reached, it was sent to author E.J.U. to review for consensus.

## 3. Results

**Figure 1:**
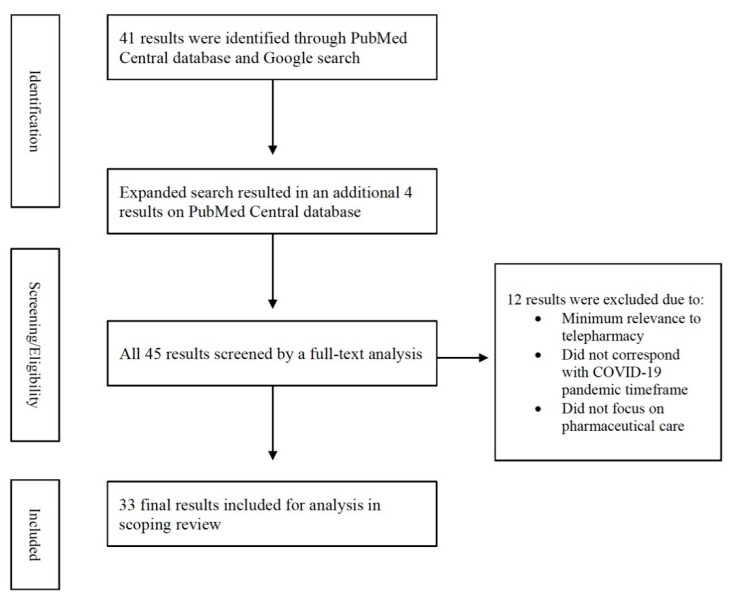
Article Selection Flowchart.

The initial search in the Spring of 2021 identified 41 articles and of these, 11 articles were excluded because they did not correspond with the COVID-19 pandemic timeframe, did not focus on pharmaceutical care or had minimal relevance to telepharmacy. After the initial analysis, a further search was conducted in the Summer of 2021 to determine if any new articles were published. This resulted in an additional four articles, of which one was excluded as per the exclusion criteria above. Figure 1 describes the selection of the studies included in this review.

A total of 33 articles were included, of which 12 were expert opinion articles, 12 were case studies and original research, and 9 were review articles (Table 1). Ten articles were from North America, seven were from the Middle East, six were from Europe, one was from Africa, six were from Asia, three were from Australia and New Zealand, and one was from The Commonwealth.

### 3.1. What Were the Various Telepharmacy Initiatives Implemented?

The most commonly observed telepharmacy initiatives were virtual consultations, home delivery of medicines and patient education based on case studies and original research articles [35,37,38,39,41,42,43,45,46,47,48]. At least one case study from a cancer institute reported offering telepharmacy to patients at-risk [33]. These initiatives are in parallel with the expert opinion articles that also suggested virtual consultations, monitoring patients’ chronic illness, optimizing medication use and patient education as part of their telepharmacy initiatives [20,25,26,29,30,31]. A network of 19 hospitals in China developed a “cloud-pharmacy care” so that patients could consult with pharmacists using texts and the internet [27,34]. Similarly, in Spain, the hospital pharmacy services adapted to use information on communication technologies [35]. This is similar to the opinion articles that suggested the use of mobile applications for providing care [21,22,23]. Another hospital in China introduced “zero-contact pharmaceutical care” using online medication consultation [39]. An institutional response to the pandemic was also implemented in the Kingdom of Saudi Arabia by adapting new e-tools such as a business WhatsApp and webpage portals [37]. In another review article from Sri Lanka, they reported the use of teleprescriptions along with videoconferencing and audio consultations [44]. In the United States, state pharmacy boards and the U.S. > Department of Health and Human Services temporarily modified requirements for telemedicine, including telepharmacy, by allowing videoconferencing that is not fully compliant with HIPAA rules [32,38]. Traynor recommended making changes to federal law to provide telehealth, including telepharmacy, and to be reimbursed for it [30]. A case study from one hospital in the U.S. reported the use of Microsoft Teams for patient education and counseling [38]. Another case study explained the use of credentialing pharmacists through various learning modules for telehealth [32]. Studies also mentioned taking verbal consent from patients for telepharmacy visits before the start of consultation [35,36]. Furthermore, review articles mentioned online discussion boards and social networking sites that could be used as initiative tactics but were not mentioned in the case studies or original research articles [49]. Bukhari et al. reported the use of hotline numbers in New Zealand for phone consultations and prescription orders, the use of mail, fax, and e-mails for prescriptions, use of mobile applications for home delivery of medicines in China and Colombia, and providing triaging and basic consultation using telepharmacy in Pakistan [24]. 

### 3.2. What Were the Challenges Faced by Pharmacies When Implementing Telehealth Initiatives?

Experts suggested technology and privacy (HIPAA compliance) would pose barriers to successful implementation of telepharmacy [25,26]. It was also thought that elderly patients and patients of lower socioeconomic status would benefit most from telepharmacy; however, it was anticipated that these populations tend to have low digital literacy and may be unable to adequately navigate the technology [28]. Additional concerns were loss of privacy and confidentiality and that the most accessible teleconferencing platforms were not secure enough to be HIPAA-compliant [20,21,23]. Another potential challenge noted by experts was the need for more time to conduct telepharmacy [26]. Other challenges included decreased quality of care and doctor–patient relationships, potential for telepharmacists not to be as engaged as in-person pharmacists, deficient reimbursement policies and poor control of controlled-substance prescriptions [20,21,22,24]. Expert opinion from developing countries raised the concern of the lack of affordable and user-friendly platforms to support telepharmacy and the lack of healthcare policies to implement telepharmacy [23]. 

The available case studies reflect many of the challenges hypothesized by expert opinions. It was seen that older generations had more difficulty participating in telepharmacy visits, and there were populations—elderly patients and those of lower socioeconomic status—with limited access to technology [32,39,47]. Telepharmacy visits also require additional resources for scheduling appointments, familiarizing patients with technology and troubleshooting technological issues [32,35,47]. The challenges aforementioned, along with obtaining consent before the visit, can increase the time for telepharmacy visits [32,35]. The privacy issues that were uncovered included digital privacy as well as challenges in finding the physical space for pharmacists to conduct private teleconsultations [43,47,49]. Decreased quality of care was seen in the form of increased dispensing and filling errors by pharmacies utilizing remote services and the inability to adapt assessment tools for telepharmacy usage [33,35,37,42]. One of the case studies from the Kingdom of Saudi Arabia reported the lack of a national policy as well as practices and processes at healthcare organizational levels as challenges in implementing telepharmacy [37]. A case study from 54 Commonwealth countries reported issues with the internet, social isolation, difficulty keeping a regular schedule, difficulty accessing tools, lack of physical workspace and difficulty communicating with co-workers as challenges to telepharmacy [40]. Reimbursement issues were also noted by one case study from Bangladesh [47]. A challenge not noted by expert opinion was limited legislation allowing implementation of telepharmacy; this was also seen in the literature review articles [37,41,47]. The literature reviews also discovered that pharmacists lacked appropriate training related to disaster management and had difficulty navigating the COVID-19 pandemic [46].

### 3.3. What Were the Strategies Used by Pharmacies to Overcome the Challenges?

Many of the strategies used to overcome the challenges were centered around assisting patients with the transition to telepharmacy. Experts recommended building trust with patients for efficient telepharmacy visits and protecting privacy by obtaining patient consent before the telepharmacy visit [20,31]. In line with expert opinion, pharmacies spent time with patients ahead of a virtual visit, explaining the process and providing education on how to use the new platforms [26]. Pharmacies prepared for patient visits by identifying patient preferences and identifying patients who require intensive pharmaceutical care and thus determining the patients who would benefit most from telepharmacy versus in-person visits [26,41]. Additionally, pharmacists also obtained information from patients to optimize appointment time [33]. It was also seen that governmental interventions, such as relaxation of regulations and messaging to relieve panic and tension, helped overcome some of the challenges [25,29,47]. A review article from Sri Lanka recommended the need for appropriate legislation and prescribing protocols, especially for controlled medications [44]. Case studies also suggested providing training to pharmacists for emergency preparedness and providing educational sessions to the public to reduce telepharmacy skepticism [40,43,49]. Other strategies included developing appropriate communication protocols with onsite and offsite team members [22]. Killeen et al. recommended using a standard disclaimer statement in virtual visits, recording patients’ satisfaction with the virtual platform or conducting a risk-benefit assessment of consultation to determine future use, ensuring insurance coverage of telepharmacy, offering phone calls vs. video chat for those with reduced digital literacy and offering Wi-Fi video instead of using cellular data [28,31]. 

### 3.4. What Were Some of the Innovative Methods Used by Pharmacies to Implement Telepharmacy?

Innovative methods arose to enhance the implementation of telepharmacy during the COVID-19 pandemic and life thereafter. The idea of a “digital health ecosystem”—where clients, providers, healthcare institutions and digital healthcare devices are interconnected in a digital health environment with the goal to improve the well-being of individuals and families — developed [44]. Collectively, expert opinion and guideline articles mentioned that innovation of applications such as the use of an Electronic Medical Record (EMR) system or APOmondo can serve as portals to ensure security and patient confidentiality [21,28,32]. APOmondo was created in April 2020 to serve as a free telepharmaceutical portal to provide personal care for patients [21]. A similar application called SEHA was created by the Kingdom of Saudi Arabia to provide visual medical consultations [37]. A case study from the U.S. reported developing patient resources to help set up and prepare the patient for a telepharmacy visit [32]. Resources were also created by professional associations, regulatory bodies and universities to assist healthcare professionals in conducting virtual consultations [26,40]. One article also reported the use of a geriatric-specific telemedicine consultation device known as GeriMedRisk [20]. These articles also emphasized appropriate legislation to ease limitations on the use of telepharmacy and expand approvals of billing codes [25,29]. Case studies and original research articles similarly mentioned innovation of applications such as WeChat, one of the largest social communication mobile platforms in China, to provide relevant pharmaceutical care [34,39]. A case study from Spain reported the use of integrating geo-location for efficient delivery of medicines [35]. Research from Spain and the United Arab Emirates reported using a telephone survey to measure patient satisfaction and evaluating the effectiveness of telepharmacy by examining the rate of dispensing errors and the number and nature of pharmacists’ interventions [41,42]. Another study from the Netherlands reported increased use of online patient education tools and online refill prescriptions [43]. Lastly, review articles focused on proper initiatives such as contacting legislation and policy makers to enhance policies to aid pharmacists to play an essential role in telepharmacy [30,36]. The use of telepharmacy can have an increased return on investment with improved productivity and efficiency, patient compliance to telepharmacy visits and can advance the practice [22,30,37]. Additionally, increased use of telepharmacy can also increase the use of electronic prescriptions and online ordering of pharmaceutical products, thus eventually resulting in safe dispensing and distribution of medications [23]. In the long term, experts recommended educating federal and state policymakers on pharmacies’ fair and equitable access to programs and opportunities to advance the profession and recognize pharmacists as healthcare providers [25,47,48]. 

## 4. Discussion

The major aim of this review was to understand how telehealth was used in pharmacies during the pandemic, with the goal to establish guidance for pharmacies trying to implement telepharmacy. Specifically, the review examined how telepharmacy was implemented, the challenges in implementation, strategies used to overcome the challenges and the innovative methods used by pharmacies to implement telepharmacy. Within the short span after the emergence of COVID-19, the health care system adapted to the use of telemedicine, including telepharmacy, to introduce new initiatives and to anticipate and overcome the challenges. While virtual consultations, remote monitoring, and e-prescriptions became quite common, the review also showed the lack of access to digital devices for telepharmacy by individuals who would benefit the most from it. This was identified as one of the barriers in implementing telepharmacy prior to COVID-19, as projected by guidelines and expert opinions. The review also showed that while countries such as the United States relaxed privacy legislation such as HIPAA for the easy implementation of telepharmacy, there were other countries that lacked a national policy for telepharmacy and telemedicine. It is also significant to discuss how these policies will change once the COVID-19 pandemic ends. Expert opinion articles emphasized the importance of adapting the policies to accommodate telepharmacy, and at least one article discussed the need for reimbursements for telepharmacy. 

The review also demonstrated the challenges in implanting telepharmacy, some expected and some unexpected. For example, the increase in time to complete a telepharmacy visit was unexpected along with the additional resources needed to complete it. However, inequity in reaching out to all populations with telepharmacy due to lack of digital devices was expected. Another unexpected issue with telepharmacy during COVID-19 was that pharmacists were also working remotely from home and hence did not have a private space or stable internet to communicate with the patients. Though there were challenges, pharmacists also adapted fast to the new requirements. However, the review also shows the need for credentialing pharmacists in telepharmacy and in disaster management. 

The enterprising characteristic of healthcare systems was also evident from the reviews. Several mobile platforms were developed quickly, and healthcare systems also adapted them at a fast pace. Different countries developed their own mobile applications, probably to suit the culture and needs of their patients. However, these entrepreneurial platforms also need support in the form of legislation.

### 4.1. Future Steps

Going forward, there are some steps that need to be taken to ensure that telepharmacy will remain a meaningful service. Pharmacists and pharmacy associations need to advocate for more permanent legislation to continue the use of telepharmacy and to increase access to it. Pharmacy organizations or pharmacy entrepreneurs may need to take steps to develop robust platforms that can be used by pharmacies and patients to communicate with each other, especially for community pharmacies. This can be similar to the EMR system created for healthcare systems. Education regarding telepharmacy is essential, not only for pharmacists but also for patients to solidify its use. Formal continuing education programs will help pharmacists stay up to date with current telepharmacy practices. Further studies are needed to examine the reimbursement for telepharmacy consultations, especially for chronic disease management. Likewise, the impact of telepharmacy on patient safety and outcomes, workload, morale and attrition on pharmacy staff, public perception and access of technology, as well as implementation of telepharmacy via social media, are important topics that merit further investigation in the future. 

### 4.2. Limitations

A few limitations of the study include the potential for missing some articles due to the fact that this is a scoping review, not a systematic review. The review only includes articles after the outbreak of the coronavirus. In addition, the challenges mentioned in telehealth implementation can differ once the pandemic is under control. Therefore, applicability may be limited after the pandemic period.

## 5. Conclusions

As a result of the COVID-19 pandemic, the use of telepharmacy increased to provide pharmaceutical care and counseling services to patients despite geographical area and other posed implementation challenges. However, the study also revealed the steps that can be taken by pharmacy organizations, payers, and pharmacy entrepreneurs in leveraging the convenience of telepharmacy.

## Figures and Tables

**Table 1:** List of included articles in the review.

	Article Reference, Title, and Country	Key Points
	Expert Opinion	
[20]	Pharmacist intervention amid the coronavirus disease 2019 (COVID-19) pandemic: from direct patient care to telemedicine. Canada	Increased demand for telepharmacy; Privacy and confidentiality concerns; Patient consent; Building trust with the patient; GeriMedRisk app
[21]	Opportunities of information communication technologies for providing pharmaceutical care in the COVID-19 pandemic. Bulgaria	Mobile applications such as APOmondo; Quality, reliability, and security requirements for healthcare system
[22]	Leveraging telecommuting pharmacists in the post-COVID-19 world. USA	HIPAA-compliant communication platforms; Ensuring efficiency of the telepharmacists working remotely; Telecommuting models
[23]	The shifting landscape of pharmaceutical care–during and beyond the COVID-19 pandemic. Iran	Digital information and communication technologies that are affordable and user friendly; patient confidentiality, Electronic prescriptions and online ordering
[24]	Pharmacists at the frontline beating the COVID-19 pandemic. New Zealand	New Zealand–Hotline numbers for phone consultations and prescription orders Australia–Remote dispensing China and Columbia–Home delivery Pakistan–Triaging patients for consultation
[25]	An expert shares pharmacy’s biggest COVID-19 lessons-so far. USA	Video/Phone consultations, Ease of requirements to Facetime or Zoom, fair and equitable access to telepharmacy programs should be advocated for after the pandemic.
[26]	Undertaking medication review by telehealth. Australia	Need of additional time and resources for telepharmacy-appropriate equipment, location, audio vs. audiovisual technology; Resources for pharmacists to conduct televisits by professional associations, regulatory bodies and universities
[27]	Providing pharmacy services during the coronavirus pandemic. China	WeChat, online remote pharmacy service
[28]	Innovations in practice: telepharmacy’s time has arrived. Canada	Reduced digital literacy; Risk of privacy and security; Disclaimer statement for all televisits, Record patient satisfaction to determine further use
[29]	Ambulatory care practice in the COVID-19 era: redesigning clinical services and experiential learning. USA	Telepharmacy for medication management using Facetime and Skype; Expanded approvals for telehealth, billing codes
[30]	Pharmacists turn to telehealth to meet patients’ needs. USA	Reimbursement for televisits; ASHP advocating for telepharmacy; Co-visit model with PCP
[31]	Telehealth: conducting medication reviews during COVID-19. Australia	Telehealth if patient meets eligibility criteria; Risk-benefit assessment to ensure suitability for patient; Informed consent; Ensuring insurance coverage
	**Case Studies/Original Research**	
[32]	Establishing clinical pharmacist telehealth services during the COVID-19 pandemic. USA	Coronavirus Preparedness and Response Supplemental Appropriations Act, credentialing pharmacists for telehealth; Additional time and resources needed for telepharmacy; HIPPA-compliant platform
[33]	Necessity of pharmacist-driven nonprescription telehealth consult services in the era of COVID-19. USA	Telehealth for at-risk populations; Adaptation of clinical assessment tools
[34]	Fighting against COVID-19: innovative strategies for clinical pharmacists. China	Cloud pharmacy care for patients to text and call pharmacists; WeChat App for online Pharmaceutical monitoring; multimedia health education
[35]	Pharmaceutical care to hospital outpatients during the COVID-19 pandemic: telepharmacy. Spain	Outpatient consultation services, home drug deliver using geolocation; increased need for resources
[36]	Survey on the situation of telepharmacy as applied to the outpatient care in hospital pharmacy departments in Spain during the COVID-19 pandemic. Spain	Strategic Outpatient Pharmaceutical Care Map for gradual implementation of telepharmacy throughout Spain; Has the capacity for nationwide implementation
[37]	Implementation and evaluation of telepharmacy during COVID-19 pandemic in an academic medical city in the Kingdom of Saudi Arabia: paving the way for telepharmacy. Saudi Arabia	Adopted institutional pandemic response model, Home delivery of medications followed by remote counseling using HIPPA-compliant video; Business WhatsApp; SEHA App for visual media consultations; National policy
[38]	Use of telemedicine to provide clinical pharmacy services during the SARS-CoV-2 pandemic. USA	Exercise enforcement discretion to use videoconferencing not fully compliant with HIPAA; Use of Telephones, Microsoft Teams and EMR for profile reviews, inpatient rounds, and patient education
[39]	Pharmacy administration and pharmaceutical care practice in a module hospital during the COVID-19 epidemic. China	Zero contact pharmaceutical care model for virtual consultation; WeChat App; Radio station to relieve panic and tension among public
[40]	COVID-19: needs assessment of the pharmacy profession and contributions so far across the Commonwealth. The Commonwealth	COVID-19 resources, CwPAMS app; Webinars to train pharmacists; challenges to remote working such as isolation
[41]	Implementation of a novel home delivery service during pandemic. Spain	Identifying patients who require onsite pharmaceutical care and telepharmacy; Medication delivery service; Telephone survey of patient satisfaction
[42]	. Evaluation of telepharmacy services in light of COVID-19. United Arab Emirates	Videoconferencing; Home delivery of meds; More dispensing errors; Effectiveness of telepharmacy was evaluated
[43]	Impact of the COVID-19 epidemic on the provision of pharmaceutical care in community pharmacies. Netherlands	Online education and counseling; Concerns about privacy; Webinars and educational sessions to reduce telepharmacy skepticism
	**Review Articles**	
[44]	A review of telehealth practices in Sri Lanka in the context of the COVID-19 pandemic. Sri Lanka	Videoconferencing and e-prescriptions; Reduced standard of care and patient–physician relationship; Poor control of controlled prescriptions; Privacy and cybersecurity, Deficient reimbursement; Need for national framework
[45]	Multilevel engagements of pharmacists during the COVID-19 pandemic: the way forward. Saudi Arabia, Pakistan	Webpages, message services and social networking links to respond to patient queries
[46]	Paradigm shift in practice: the role of pharmacists in COVID-19 management. Pakistan	Multimedia health education; Multidisciplinary treatments; Need for training and curriculum for disaster management practice for pharmacists
[47]	Prospect of tele-pharmacists in COVID-19 pandemic situation in Bangladesh. Bangladesh	Physician-led responsive model and telehealthcare by pharmacists
[48]	Scope of tele-pharmacists in pandemic situations of Bangladesh. Bangladesh	Responsive model, policy makers, appropriate and quality information
[49]	A systematic review of randomized controlled trials of telehealth and digital technology use by community pharmacists to improve public health. USA	Social media, smartphone mobile application, health and digital literacy, blueprint, public awareness
[50]	Pharmacists’ experience, competence and perception of telepharmacy technology in response to COVID-19. Jordan	Social media platforms, web-based survey, quality of pharmaceutical care
[51]	Development of an Online Telepharmacy Service in the Philippines and Analysis of Its Usage During the COVID-19 Pandemic. Philippines	Online telepharmacy service, alleviate fear, counseling services, Google Form
[52]	Telepharmacy: An opportunity for community pharmacists during the COVID-19 pandemic in Sub Saharan Africa. Africa	Telepharmacy models, videoconferencing, medication delivery, medication adherence
